# Assessment of the Anthelmintic Efficacy of Albendazole in School Children in Seven Countries Where Soil-Transmitted Helminths Are Endemic

**DOI:** 10.1371/journal.pntd.0000948

**Published:** 2011-03-29

**Authors:** Jozef Vercruysse, Jerzy M. Behnke, Marco Albonico, Shaali Makame Ame, Cécile Angebault, Jeffrey M. Bethony, Dirk Engels, Bertrand Guillard, Nguyen Thi Viet Hoa, Gagandeep Kang, Deepthi Kattula, Andrew C. Kotze, James S. McCarthy, Zeleke Mekonnen, Antonio Montresor, Maria Victoria Periago, Laurentine Sumo, Louis-Albert Tchuem Tchuenté, Dang Thi Cam Thach, Ahmed Zeynudin, Bruno Levecke

**Affiliations:** 1 Department of Virology, Parasitology and Immunology, Ghent University, Faculty of Veterinary Medicine, Merelbeke, Belgium; 2 School of Biology, University of Nottingham, Nottingham, United Kingdom; 3 Public Health Laboratory, Ivo de Carneri, Pemba Island, Zanzibar, Tanzania; 4 Institut Pasteur in Cambodia, Clinical Pathology Unit, Phnom Penh, Cambodia; 5 Instituto René Rachou, Fundação Oswaldo Cruz, Belo Horizonte, Minas Gerais, Brazil; 6 Department of Neglected Tropical Diseases, World Health Organization, Geneva, Switzerland; 7 National Institute for Malariology, Parasitology and Entomology, Hanoi, Vietnam; 8 Department of Gastrointestinal Sciences, Christian Medical College, Vellore, India; 9 Division of Livestock Industries, Commonwealth Scientific and Industrial Research Organisation, Brisbane, Australia; 10 Queensland Institute for Medical Research, University of Queensland, Herston, Australia; 11 Department of Medical Laboratory Sciences and Pathology, College of Public Health and Medical Sciences, Jimma University, Jimma, Ethiopia; 12 Centre for Schistosomiasis and Parasitology, Faculty of Sciences, University of Yaoundé I, Cameroon; London School of Hygiene & Tropical Medicine, United Kingdom

## Abstract

**Background:**

The three major soil-transmitted helminths (STH) *Ascaris lumbricoides*, *Trichuris trichiura* and *Necator americanus/Ancylostoma duodenale* are among the most widespread parasites worldwide. Despite the global expansion of preventive anthelmintic treatment, standard operating procedures to monitor anthelmintic drug efficacy are lacking. The objective of this study, therefore, was to define the efficacy of a single 400 milligram dose of albendazole (ALB) against these three STH using a standardized protocol.

**Methodology/Principal Findings:**

Seven trials were undertaken among school children in Brazil, Cameroon, Cambodia, Ethiopia, India, Tanzania and Vietnam. Efficacy was assessed by the Cure Rate (CR) and the Fecal Egg Count Reduction (FECR) using the McMaster egg counting technique to determine fecal egg counts (FEC). Overall, the highest CRs were observed for *A. lumbricoides* (98.2%) followed by hookworms (87.8%) and *T. trichiura* (46.6%). There was considerable variation in the CR for the three parasites across trials (country), by age or the pre-intervention FEC (pre-treatment). The latter is probably the most important as it had a considerable effect on the CR of all three STH. Therapeutic efficacies, as reflected by the FECRs, were very high for *A. lumbricoides* (99.5%) and hookworms (94.8%) but significantly lower for *T. trichiura* (50.8%), and were affected to different extents among the 3 species by the pre-intervention FEC counts and trial (country), but not by sex or age.

**Conclusions/Significance:**

Our findings suggest that a FECR (based on arithmetic means) of >95% for *A. lumbricoides* and >90% for hookworms should be the expected minimum in all future surveys, and that therapeutic efficacy below this level following a single dose of ALB should be viewed with concern in light of potential drug resistance. A standard threshold for efficacy against *T. trichiura* has yet to be established, as a single-dose of ALB is unlikely to be satisfactory for this parasite.

**Trial Registration:**

ClinicalTrials.gov NCT01087099

## Author Summary

Soil-transmitted helminths (roundworms, whipworms and hookworms) infect millions of children in (sub)tropical countries, resulting in malnutrition, growth stunting, intellectual retardation and cognitive deficits. Currently, there is a need to closely monitor anthelmintic drug efficacy and to develop standard operating procedures, as highlighted in a World Health Organization–World Bank meeting on “Monitoring of Drug Efficacy in Large Scale Treatment Programs for Human Helminthiasis” in Washington DC at the end of 2007. Therefore, we have evaluated the efficacy of a commonly used treatment against these parasitic infections in school children in Africa, Asia and South-America using a standardized protocol. In addition, different statistical approaches to analyzing the data were evaluated in order to develop standardized procedures for data analysis. The results demonstrate that the applied treatment was highly efficacious against round- and hookworms, but not against whipworms. However, there was large variation in efficacy across the different trials which warrants further attention. This study also provides new insights into the statistical analysis of efficacy data, which should be considered in future monitoring and evaluation studies of large scale anthelmintic treatment programs. Finally, our findings emphasize the need to update the World Health Organization recommended efficacy threshold for the treatment of STH.

## Introduction

The three major Soil-Transmitted Helminths (STH), *Ascaris lumbricoides* (roundworm), *Trichuris trichiura* (whipworm) and *Necator americanus/Ancylostoma duodenale* (the hookworms) are amongst the most widespread parasites worldwide. An estimated 4.5 billion individuals are at risk of STH infection and more than one billion individuals are thought to be infected, of whom 450 million suffer morbidity from their infection, the majority of who are children. An additional 44 million infected pregnant women suffer significant morbidity and mortality due to hookworm-associated anemia. Approximately 135,000 deaths occur per year, mainly due to infections with hookworms or *A. lumbricoides* [1].

The most widely implemented method of controlling STH infections is through periodic administration of anthelmintics. Rather than aiming to achieve eradication, current control programs are focused on reducing infection intensity and transmission potential, primarily to reduce morbidity and avoid mortality associated with the disease [2]. The benzimidazole (BZ) drugs, i.e. albendazole (ALB) and mebendazole, are the most widely used drugs for the control of STH. While both show broad-spectrum anthelmintic activity, for hookworms a single dose of ALB is more effective than mebendazole [3].

The scale up of chemotherapy programs that is underway in various parts of Africa, Asia and South America, particularly targeting school children, is likely to exert increasing drug pressure on parasite populations, a circumstance that is likely to favor parasite genotypes that can resist anthelmintic drugs. Given the paucity of suitable alternative anthelmintics it is imperative that monitoring programs are introduced, both to assess progress and to detect any changes in therapeutic efficacy that may arise from the selection of worms carrying genes responsible for drug resistance. The well documented occurrence of resistance to anthelmintics in nematode populations of livestock [4], highlights the potential for frequent treatments used in chemotherapy programs to select drug resistant worms. Such an eventuality threatens the success of treatment programs in humans, both at individual and community levels [5]. Although some small scale studies [6], [7], have suggested emerging drug resistance in human STH, these studies should be interpreted with some caution, since suboptimal efficacy could have been due to factors other than drug resistance. Moreover, although for the BZ drugs there are many published studies reporting the Cure Rate (CR) and the Fecal Egg Count Reduction (FECR), the two most widely used indicators for assessing the efficacy of an anthelmintic in human medicine, comparison of such studies is difficult, largely because there is no widely accepted standard operating procedure for undertaking such trials [8]. Published studies are confounded by methodological variations including treatment regimens, poor quality of drugs, differing statistical analyses used to calculate therapeutic efficacy, as well as a range of other problems in study design, such as small sample size, diagnostic methods, variation in pre-intervention infection intensities and confounding factors related to geographical locations. Such variation among studies greatly complicates direct comparison [3]. A World Health Organization-World Bank (WHO-WB) meeting on “Monitoring of Drug Efficacy in Large Scale Treatment Programs for Human Helminthiasis”, held in Washington DC at the end of 2007, highlighted the need to closely monitor anthelmintic drug efficacy and to develop standard operating procedures for this purpose. In a systematic meta-analysis of published single-dose studies, Keiser and Utzinger [8], confirmed that there was a paucity of high quality trials, and that the majority of trials were carried out more than 20 years ago. They recommended that well-designed, adequately powered, and rigorously implemented trials should be undertaken to provide current data regarding the efficacy of anthelmintics against the main species of STH. These should be designed to establish benchmarks (including standard operating procedures) for subsequent monitoring of drug resistance.

The objective of the present work was to validate a standard protocol that has been developed for monitoring efficacy of anthelmintics against STH. To give the study wide relevance, we conducted the trial in seven populations in different geographic locations in Brazil, Cameroon, Cambodia, Ethiopia, India, Tanzania and Vietnam. In each of the study sites, different epidemiologic patterns of infection prevail, including different combinations of STH. We assessed the efficacy of a single dose (400 mg) of ALB in terms of the CR and the FECR in school children between 14 and 30 days following treatment. The McMaster egg counting technique was used in a standardized fashion, with rigorous quality control. Levecke et al. [9] reported that the McMaster holds promise as a standardized method on account of its applicability for quantitative screening of large numbers of subjects. This method is the recommended method for measuring fecal egg counts (FEC) when performing FECR for the detection of anthelmintic resistance in veterinary medicine [10], [11].

## Methods

### Study sites

This study was carried out in seven different countries covering Africa (Cameroon, Ethiopia and Tanzania), Asia (Cambodia, India and Vietnam) and South-America (Brazil). However, it is important to note, that while we refer to individual countries to identify results from particular trials, we do not make any conclusions about any country as such. Here, names of countries are used only to distinguish between 7 separate trials that were conducted in 7 geographically disparate regions of the world. In total ten study sites with varying STH and treatment history were included. These seven STH endemic countries were selected because of the presence of investigator groups with previous extensive experience in the diagnosis and control of STH. Table 1 provides their specific locations (district/province/state) and treatment history. Both species of hookworms (*N. americanus* and *A. duodenale*) were present in all study sites in varying degree with the exception of Brazil where only *N. americanus* was present.

**Table 1:** The location and treatment history of the ten study sites.

Country	District/Province/State	Treatment history
**Brazil**	Minas Gerais	LSAT since 2007 (ALB)
**Cambodia**	Kratie	LSAT since 1997, last in 2007 (MBD)
**Cameroon**	Loum	LSAT (MBD/ALB) since 1999, last in 2008 (MBD)
	Yoyo	No LSAT
**Ethiopia**	Jimma	No LSAT
**India**	Vellore	LSAT, since 2001, last in 2008 (ALB)
	Thiruvanamalai	No LSAT
**Tanzania (Zanzibar)**	Pemba Island	LSAT since 1994, last in 2006 (PZQ, IVM, ALB)
**Vietnam**	Thái Nguyên	LSAT since 2005
	Tuyên Quang	No LSAT

LSAT: large scale anthelmintic treatment, MBD: mebendazole, PZQ: praziquantel, IVM: ivermectine, ALB: albendazole.

### Trial design

During the pre-intervention survey, school children aged 4 to 18 years at the different study sites were asked to provide a stool sample. For the initial sampling the aim was to enroll at least 250 infected children with a minimum of 150 eggs per gram of feces (EPG) for at least one of the STH. This sample size was selected based on statistical analysis of study power, using random simulations of correlated over-dispersed FEC data reflecting the variance-covariance structure in a selection of real FEC data sets. This analysis suggested that a sample size of up to 200 individuals (α = 0.05, power = 80%) was required to detect a 10 percentage point drop from a null efficacy of ∼ 80% (mean percentage FEC Δ per individual) over a wide range of infection scenarios. Standard power analyses for proportions also indicated that the detection of a ∼10 percentage point drop from a null cure rate required sample sizes up to 200 (the largest samples being required to detect departures from null efficacies of around 50%). Given an anticipated non-compliance rate of 25%, a sample of 250 individuals with >150 EPG pre-treatment was therefore considered necessary at each study site.

**Figure 1:**
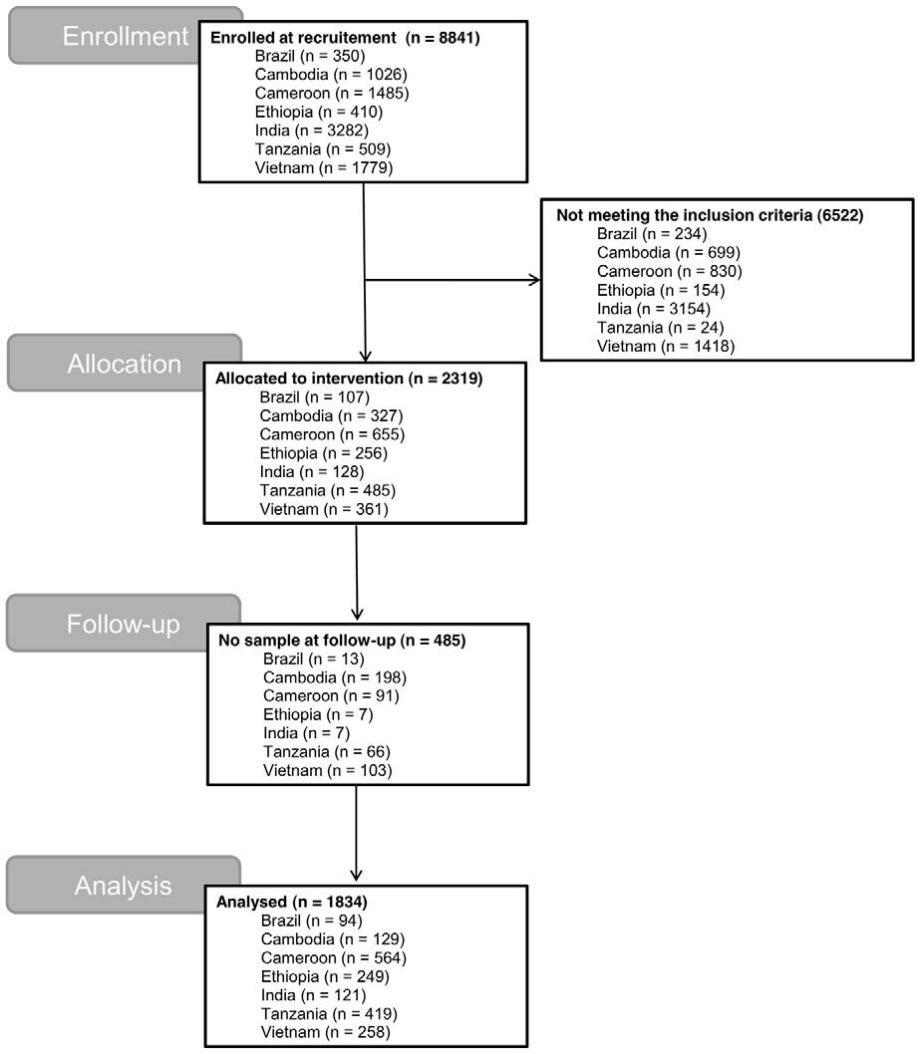
The participation, occurrence of STH and sample submission compliance for pre- and post-intervention surveys. Subjects who were not able to provide a sample for the follow-up, or who were experiencing a severe current medical condition or had diarrhea at the time of the first sampling were excluded from the trial.

Fecal samples were processed using the McMaster technique (analytic sensitivity of 50 EPG) for the detection and the enumeration of infections with *A. lumbricoides*, *T. trichiura* and hookworms [9]. None of the samples were preserved. Samples which could not be processed within 24 hours were kept at 4°C. A single dose of 400 mg ALB (Zentel) from the same manufacturer (GlaxoSmithKline Pharmaceuticals Limited, India) and same lot (batch number: B.N°: L298) was used at all trial sites. No placebo control subjects were included in the trial for ethical and operational reasons. Between 14 to 30 days after the pre-intervention survey, stool samples were collected from the treated subjects and processed by the McMaster technique. All of the trials were carried out in a single calendar year (2009). Subjects who were unable to provide a stool sample at follow-up, or who were experiencing a severe concurrent medical condition or had diarrhea at time of the first sampling, were excluded from the study. The participation, the occurrence of STH and sample submission compliance for pre- and post-intervention surveys are summarized in Figure 1.

### The McMaster counting technique

The McMaster counting technique (McMaster) was based on the modified McMaster described by the Ministry of Agriculture, Fisheries and Food (UK; 1986) [12]. Two grams of fresh stool samples were suspended in 30 ml of saturated salt solution (density = 1.2). The suspension was poured three times through a wire mesh to remove large debris. Then 0.15 ml aliquots were added to each of the 2 chambers of a McMaster slide. Both chambers were examined under a light microscope using a 100x magnification and the FEC for each helminth species was obtained by multiplying the total number of eggs by 50.

### Statistical analysis

The efficacy of the treatment for each of the three STH was evaluated qualitatively based on the reduction in infected children (CR) and quantitatively based on the reduction in fecal egg counts (FECR). The outcome of the FECR was calculated using three formulae. The first two formulae were based on the mean (arithmetic/geometric) of the pre- and post-intervention fecal egg count (FEC) ignoring the individual variability, whereas the third formula represented the mean of the reduction in the FEC per subject. The latter is the only quantitative indicator of efficacy for which the importance of confounding factors can be assessed by statistical analysis.



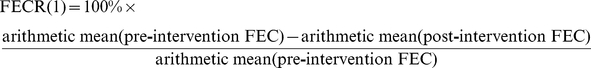


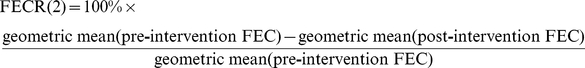


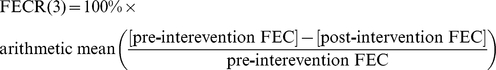
The CR and the FECR (1-3) outputs were calculated for the different trials, both sexes, age classes (A: 4–8 years; B: 9–13 years and C: 14–18 years) and for the level of egg excretion intensity at the pre-intervention survey. These levels corresponded to the low, moderate and high intensities of infection as described Montresor et al. [13] For *A. lumbricoides* these were 1–4,999 EPG, 5,000–49,999 EPG and >49,999 EPG; for *T. trichiura* these levels were 1–999 EPG, 1000–9,999 EPG and >9,999 EPG; and for hookworms these were 1–1,999 EPG, 2,000–3,999 EPG and >3,999 EPG, respectively.

In addition, the robustness of the three FECR formulae was explored by comparing the FEC reduction rate obtained from all samples containing STH and those obtained from samples containing more than 150 EPG as recommended in the anthelmintic resistance guidelines of the World Association for the Advancement of Veterinary Parasitology [9]. Finally, putative factors affecting the CR and the FECR (3) were evaluated. For the CR, generalized linear models (binomial error) were built with the test result (infected /uninfected) as the outcome, ‘trial’ (7 levels: trials in Brazil, Cambodia, Cameroon, Ethiopia, India, Tanzania and Vietnam) and ‘sex’ (2 levels: female and male) as factors, and ‘age’ and the log transformed pre-intervention FEC as covariates. Full factorial models were evaluated by the backward selection procedure using the likelihood ratio test of χ^2^. Finally, the CR for each of the observed values of the covariate and factor was calculated based on these models (The R Foundation for Statistical Computing, version 2.10.0 [14]). For analysis of the data from FECR (3), non-parametric methods were used, because models based on parametric statistics, even with negative binomial error structures, or based on transformed data would not converge satisfactorily as a consequence of the high proportion of FEC with zero EPG. Hence, the impact of the factors ‘trial’ and ‘sex’ were assessed by the Kruskal-Wallis test (for more than 2 group comparisons) and the Mann-Whitney U test, respectively. The correlation between the outputs of FECR (3) and the covariates (age and pre-intervention FEC) was estimated by the Spearman rank order correlation coefficient (SAS 9.1.3, SAS Institute Inc.; Cary, NC, USA).

### Ethics statement

The overall protocol of the study was approved by the Ethics committee of the Faculty of Medicine, Ghent University (Nr B67020084254) and was followed by a separate local ethical approval for each study site. For Brazil, approval was obtained from the Institutional Review Board from Centro de Pesquisas René Rachou (Nr 21/2008), for Cambodia from the National Ethic Commitee for Health Research, for Cameroon from the National Ethics Committee (Nr 072/CNE/DNM08), for Ethiopia from the Ethical Review Board of Jimma University, for India from the Institutional Review Board of the Christian Medical College (Nr 6541), for Tanzania (Nr 20) from the Zanzibar Health Research Council and the Ministry of Health and Social Welfare, for Vietnam by the Ministry of Health of Vietnam. An informed consent form was signed by the parents of all subjects included in the study. This clinical trial was registered under the ClinicalTrials.gov Identifier NCT01087099.

## Results

### The cure rate (CR)

Overall, the highest CRs were observed for *A. lumbricoides* (98.2%), followed by hookworm (87.8%) and *T. trichiura* (46.6%). However, as shown in Table 2, the CRs varied across the different trials, age classes and pre-intervention FEC levels. The differences in CRs between trials were most pronounced for *T. trichiura*, ranging from 21.0 (Tanzania) to 88.9% (India). The *T. trichiura* CRs of 100% for the trials in Brazil and Cambodia are not considered here as they were based on only 1 and 2 individuals, respectively. For hookworms and *A. lumbricoides*, the CRs varied from 74.7 (India) to 100% (Vietnam) and from 96.4 (Tanzania) to 99.3% (Ethiopia and Cameroon), respectively. The CRs for *A. lumbricoides* in Cambodia (100%) and India (95.2%) are not considered here as they were based on fewer than 50 individuals. The CRs increased over the three age classes (*A. lumbricoides*: 95.8 to 100%; *T. trichiura*: 44.7 to 54.1%), except for hookworms where the CRs ranged from 86.1 to 88.3, and then to 87.5%. For each of the three STH, there was a decline in the CR with increasing levels of infection intensities at the pre-intervention survey. The largest drop was observed for *T. trichiura*, which decreased from 53.9 to 12.5%. For the two other STH, the drop in the CR was less pronounced, ranging from 88.6 to 76.9% for hookworms and only from 98.3 to 95% for *A. lumbricoides*. The observed differences between sexes were negligible for all three STH.

**Table 2:** The cure rate (CR) for treatment with a single dose of albendazole against soil-transmitted helminths.

	*A. lumbricoides*			*T. trichiura*		Hookworms	
	n	CR (%)		N	CR (%)	n	CR (%)
**Country**							
Brazil	50	98.0		1*	100	52	88.5
Cambodia	5*	100		2*	100	127	87.4
Cameroon	298	99.3		386	47.4	140	87.1
Ethiopia	151	99.3		105	85.7	91	98.9
India	21*	95.2		18*	88.9	95	74.7
Tanzania	279	96.4		396	21.0	349	86.8
Vietnam	148	98.6		138	81.2	58	100
**Age class**							
A (4–8)	215	95.8		219	44.7	173	86.1
B (9–13)	669	98.8		753	46.3	643	88.3
C (14–18)	68	100		74	54.1	96	87.5
**Sex**							
Female	462	98.1		503	48.5	393	89.1
Male	490	98.4		543	44.8	519	86.9
**Pre-intervention infection intensity**							
Low	662		98.3	823	53.9	859	88.6
Moderate	270		98.1	215	19.5	40	75.0
High	20		95.0	8	12.5	13	76.9
**Total**	**952**		**98.2**	**1046**	**46.6**	**912**	**87.8**

*Due to the low number of infected subjects (<50), the trials conducted in these countries were excluded from further analysis.

**Figure 2:**
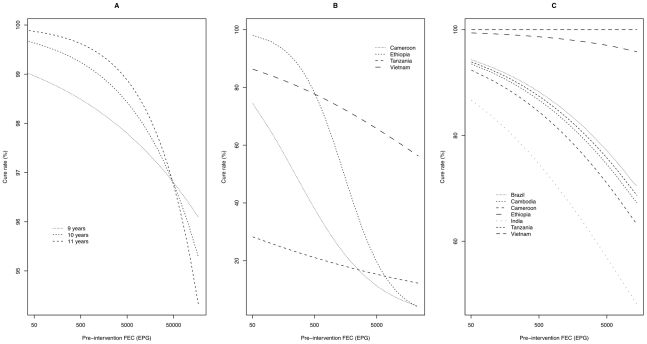
The estimated impact of age, fecal egg count and trial on the cure rate. The estimated impact of age, fecal egg count (FEC) and trial (country) on the cure rate of *A. lumbricoides* (A), *T. trichiura* (B) and hookworms (C) derived from a generalized linear models (binomial error).

Differences in CR by trial, age and pre-intervention FEC are illustrated in Figure 2. The variability in the CR of the three parasites was significantly associated with these three factors (predictive value >75%). The pre-intervention FEC was probably the most important as it had a considerable effect on the CR of *A. lumbricoides* (χ^2^_1_ = 4.14, *p*<0.05), *T. trichiura* (χ^2^_1_ = 66.3, *p*<0.0001) and hookworms (χ^2^_1_ = 11.9, *p*<0.001). Age only contributed to variation in the CR of *A. lumbricoides* (χ^2^_1_ = 6.8, *p*<0.01). Differences among the trials (countries) in the CR were observed for *T. trichiura* (χ^2^_3_ = 33.8, *p*<0.0001) and hookworms (χ^2^_6_ = 35.1, *p*<0.0001), but not for *A. lumbricoides*. In addition, there was an interaction between the pre-intervention FEC for *A. lumbricoides* (χ^2^_1_ = 4.7, *p*<0.05) and for *T. trichiura* (χ^2^_3_ = 18.4, *p*<0.0005) with age and trial (country) respectively (lines cross one another). The impact of pre-intervention FEC on the CR of *A. lumbricoides* was more pronounced for older individuals than younger ones. For *T. trichura* the effect of pre-intervention FEC varied considerably across the trials conducted in the different countries, particularly for the trial in Ethiopia where the CR dropped from almost 100 to nearly 0% as the pre-intervention FEC increased.

### Comparison of different formulae for assessing FECR

**Figure 3:**
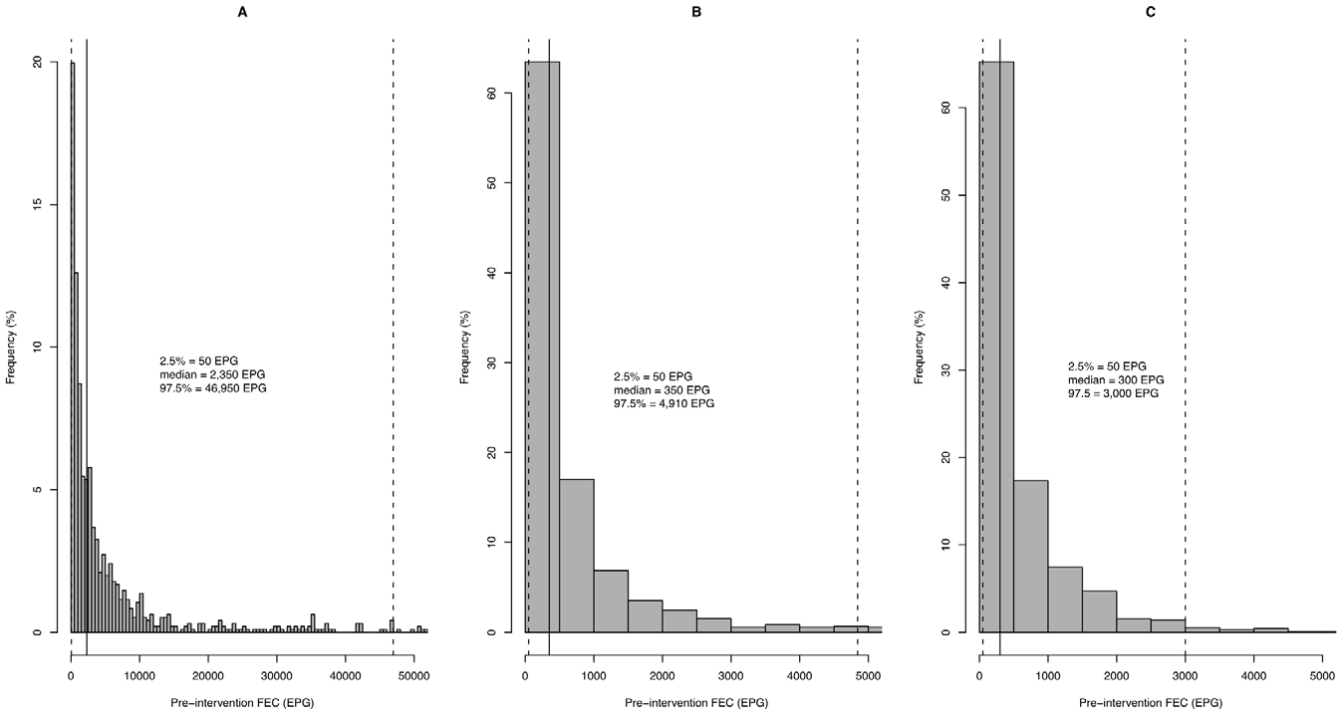
The distribution of the fecal egg count of soil-transmitted helminths. The distribution of the fecal egg count (FEC) of *A. lumbricoides* (A), *T. trichiura* (B) and hookworms (C). The absolute values of the median, the 2.5^th^ and 97.5^th^ percentile (%) are provided. In addition, these values are represented as solid (median) and dashed lines (2.5 and 97.5%).

The pre-intervention FEC for the different STH ranged from 50 to 170,500 EPG for *A. lumbricoides* (arithmetic mean = 6877 EPG), from 50 to 23,200 EPG for *T. trichiura* (arithmetic mean = 824 EPG) and from 50 to 13,800 EPG for hookworm (arithmetic mean = 650 EPG). The data in Table 3 show that there was considerable variation in the arithmetic means of the FEC from the trial groups in the 7 participating countries for each of the three STH species. As illustrated in Figure 3, pre-intervention FEC were highly aggregated among the subjects, and high FEC were only observed in relatively few subjects.

**Table 3:** The arithmetic means of fecal egg counts of soil-transmitted helminths across the seven trials (countries).

	*A. lumbricoides*		*T. trichiura*		Hookworms	
	N	Arithmetic mean(min-max)	n	Arithmetic mean(min-max)	n	Arithmetic mean(min-max)
Brazil	50	9230(100–51250)	1	50	52	617(50–2900)
Cambodia	5	1420(50–6600)	2	175(50–300)	127	585(50–4400)
Cameroon	298	12085(50–170500)	386	1023(50–23200)	140	567(50–13800)
Ethiopia	151	3443(100–62500)	105	420(100–5200)	91	326(100–1650)
India	21	2927(50–13100)	18	305(50–1100)	95	662(100–3000)
Tanzania	279	4279(50–55750)	396	924(50–6650)	349	867(50–6950)
Vietnam	148	4741(50–53000	138	371(50–4600)	58	205(50–2500)
Total	952	6877**(50–170500)**	1046	824**(50–23200)**	912	650**(50–13800)**

The FEC reduction rate calculated using all three formulae (based on FECR 1-3) in turn for *A. lumbricoides*, *T. trichiura* and hookworms across the 7 trials (countries), age classes, sexes and pre-intervention infection intensities are summarized in Table 4. Overall, the FEC reduction rate for FECR(1) was the highest for *A. lumbricoides* (99.5%), followed by hookworm (94.8%) and *T. trichiura* (50.8%). However, there was considerable variation in the FEC reduction rate among the 7 trials, age classes and infection intensities at pre-intervention survey. For *A. lumbricoides*, the FEC reduction rate remained roughly unchanged over these variables, only ranging from 97.8 to 100%. This contrasts with *T. trichiura*, for which the FEC reduction rate differed between the trials (from 39.2 [Cameroon] to 92.4% [Ethiopia]), age classes (from 45.4 [B] to 62.7% [A]) and pre-intervention infection intensity (from 40.0 [high] to 58.7% [moderate]). There was no difference in the FEC reduction rate between the sexes. For hookworms, only small differences in the FEC reduction rate were observed between the trials, ranging from 87.1 [India] to 100% [Vietnam]. However, there were only negligible differences between the age classes (from 94.7 [B] to 96.4% [C]).

**Table 4:** Fecal egg count reduction across different countries, age classes, sexes and pre-intervention intensities.

	*A. lumbricoides*				*T. trichiura*				Hookworms			
	n	FECR(1)(%)	FECR(2)(%)	FECR(3)(%)	n	FECR(1)(%)	FECR(2)(%)	FECR(3)(%)	n	FECR(1)(%)	FECR(2)(%)	FECR(3)(%)
**Country**												
Brazil	50	100.0	100.0	100.0	1*	100.0	98.0	100.0	52	97.5	99.6	97.8
Cambodia	5*	100.0	99.6	100.0	2*	100.0	99.2	100.0	127	97.6	99.5	96.6
Cameroon	298	99.2	100.0	26.0	386	39.2	93.0	34.7	140	93.0	99.2	91.9
Ethiopia	151	100.0	99.9	100.0	105	92.4	99.1	93.1	91	99.7	99.6	99.9
India	21*	98.9	99.8	99.7	18*	74.5	98.9	92.1	95	87.1	99.2	81.6
Tanzania	279	100.0	99.9	99.9	396	52.0	82.6	−36.2	349	95.3	99.6	92.6
Vietnam	148	100.0	99.9	99.9	138	92.3	98.8	86.4	58	100.0	99.3	100.0
**Age class**												
A (4–8)	215	98.9	100.0	−2.7	219	62.7	94.0	32.2	173	94.8	99.5	95.2
B (9–13)	669	99.8	99.9	100.0	753	45.4	93.8	16.3	643	94.7	99.5	92.3
C (14–18)	68	100.0	100.0	100.0	74	59.6	94.7	45.4	96	96.4	99.5	97.0
**Sex**												
Female	462	100.0	99.9	99.9	503	50.3	94.6	9.3	393	95.2	99.5	94.2
Male	490	99.0	99.9	54.9	543	51.2	93.2	33.2	519	94.5	99.5	92.8
**Pre-intervention infection intensity**												
Low	662	97.8	99.9	66.6	823	40.2	94.3	11.9	859	93.9	99.4	93.2
moderate	270	100.0	100.0	100.0	215	58.7	92.2	58.7	40	97.6	99.9	97.1
High	20	99.5	100.0	99.6	8	40.0	88.6	40.1	13	95.9	99.9	96.4
**Total**	**952**	**99.5**	**99.9**	**76.8**	**1046**	**50.8**	**93.9**	**21.7**	**912**	**94.8**	**99.5**	**93.4**

FECR(1): group based and arithmetic mean; FECR(2): group based and geometric mean; FECR(3): individual based and arithmetic.

*Due to the low number of infected subjects (<50), the trials conducted in these countries were excluded from further analysis.

Compared to the results of FECR (1), the outputs of FECR (2) resulted in higher values for all three STH, except for *A. lumbricoides* where the FEC reduction rate already showed a ceiling effect (100%). Considerable variation in the FEC reduction rate (FECR (2)) occurred with *T. trichiura* among the trials (from 82.6 [Tanzania] to 99.1% [Ethiopia]) and pre-intervention infection intensity (from 88.6 [high] to 94.3% [low]). For hookworms, the differences between the trials were virtually negligible, all indicating a potent effect just short of the maximum 100% (FECR (2) >99.3%).

The results of FECR (3) mostly yielded comparable or lower values than those from FECR (1). The low values (sometimes negative) can be explained by subjects for whom the post-intervention FEC exceeded the pre-intervention FEC. These subjects contributed to a negative FEC reduction rate which had a significant impact on the final FEC reduction rate calculated with FECR (3). This became apparent in the FEC reduction rate for *A. lumbricoides,* where a Cameroonian male subject of 7 years with a pre-intervention FEC of 100 and a post-intervention FEC of 22,050 EPG, contributed markedly to lowering the overall values for the data-set from the trial in Cameroon (FECR (1): 99.2%; FECR (3): 26.0%). This lowering of FECR (3) compared to FECR (1) for *A. lumbricoides* also occurred with age class A (FECR (1): 98.9%; FECR (3): −2.7%) and the low pre-intervention infection intensity level (FECR (1): 97.8%; FECR (3): 66.6%), but not for the remaining variables. The number of negative individual FEC reduction rates, and the magnitude of the difference between pre- and post-intervention FEC, both contributed to the discrepancies found for *T. trichiura* (176 subjects) and hookworms (10 subjects).

### Robustness of FECR formulae

Table 5 summarizes the FEC reduction rates restricted to samples of more than 150 EPG indicating that the results of FECR (1) and FECR (2) remained roughly unchanged. The values from FECR (3) increased and were mostly comparable with those obtained by FECR (1). This change in the results of FECR (3) is due to the exclusion of negative individual FEC reduction rates which mostly occurred among the subjects with low pre-intervention FEC (see also Table 4). Differences of more than 5% between the results of FECR (3) and FECR (1) were limited to *T. trichiura* (country: Cameroon, India, Tanzania and Vietnam; age class: A and C).

**Table 5:** Fecal egg count reduction for samples with a pre-intervention FEC of more than 150 EPG.

	*A. lumbricoides*				*T. trichiura*				Hookworms			
	n	FECR(1)(%)	FECR(2)(%)	FECR(3)(%)	n	FECR(1)(%)	FECR(2)(%)	FECR(3)(%)	n	FECR(1)(%)	FECR(2)(%)	FECR(3)(%)
**Country**												
Brazil	47*	100.0	100.0	100.0	0*	_	_	_	46*	97.5	99.6	97.5
Cambodia	1*	100.0	100.0	100.0	1*	100.0	99.7	100.0	100	97.7	99.6	96.7
Cameroon	266	99.8	100.0	100.0	233	39.9	93.4	50.4	71	93.6	99.5	95.1
Ethiopia	145	100.0	99.9	100.0	72	92.3	99.2	92.6	66	99.6	99.7	99.8
India	17*	98.9	99.9	99.6	11*	72.0	99.1	87.0	83	87.8	99.3	84.2
Tanzania	266	100.0	100.0	99.9	325	58.3	86.6	36.4	281	95.4	99.7	93.1
Vietnam	130	100.0	99.9	99.9	71	93.1	99.2	88.0	19*	100.0	99.7	100.0
**Age class**												
A (4–8)	196	99.9	100.0	99.8	153	65.1	94.8	57.2	130	94.7	99.6	94.4
B (9–12)	613	99.8	100.0	99.9	515	48.4	94.1	51.8	460	94.9	99.6	93.2
C (13–18)	63	100.0	100.0	100.0	45	60.2	94.4	46.4	76	96.4	99.6	97.1
**Sex**												
Female	428	100.0	100.0	99.9	343	54.0	94.7	57.7	286	95.2	99.6	93.8
Male	444	99.7	100.0	99.9	370	53.0	93.8	48.0	380	94.8	99.6	94.0
**Pre-intervention infection intensity**												
Low	582	99.9	99.9	99.9	490	49.0	95.1	50.2	613	94.1	99.6	93.6
Moderate	270	100.0	100.0	100.0	215	58.7	92.2	58.7	40	97.6	99.9	97.1
High	20	99.5	100.0	99.6	8	40.0	88.6	40.1	13	95.9	99.9	96.4
**Total**	**872**	**99.9**	**100.0**	**99.9**	**713**	**53.5**	**94.3**	**52.7**	**666**	**95.0**	**99.6**	**93.9**

FECR(1): group based and arithmetic mean; FECR(2): group based and geometric mean; FECR(3): individual based and arithmetic.

*Due to the low number of infected subjects (<50), the trials conducted in these countries were excluded from further analysis.

### Factors associated with FECR

The assessment of putative factors affecting the results from FECR (3) was restricted to samples containing more than 150 EPG. Due to the limited variation in the FEC reduction rates (FECR (3)) of *A. lumbricoides* across the different variables, this species was not analyzed further. Also, because of the limited number of infected subjects (<50), the trials in Brazil, Cambodia and India were excluded from analyses of *T. trichiura*. For hookworms, and for the same reasons, subjects from the trials in Brazil and Vietnam were not included. Significant differences in the FEC reduction rates between the trials were found for both *T. trichiura* (χ^2^_3_ = 117.3, *p*<0.0001) and hookworms (χ^2^_4_ = 20.2, *p* = 0.0005). High pre-intervention FEC of *T. trichiura* yielded lower FEC reduction rates (3) (R_s_ = −0.18, n = 701, p<0.0001), but this was not found for hookworm (R_s_ = −0.04, n = 601, p = 0.34). In addition, there was an interaction between the pre-intervention FEC of *T. trichiura* and trial (country), reflected in the negative correlations in the trials in Cameroon (R_s_ = −0.28, n = 233, p<0.0001), and Ethiopia, (R_s_ = −0.34, n = 72, p = 0.0034), but a positive correlation for the trial in Tanzania (R_s_ = +0.28, n = 325, p<0.0001) and a non-significant correlation for the trial in Vietnam (R_s_ = −0.07, n = 71, p = 0.58). Host sex and age did not contribute significantly to variation of the results of (FECR (3)) in any of the STH examined.

## Discussion

To our knowledge, the present study is the first to evaluate drug efficacy for STH in school children across different endemic regions using a protocol which was standardized in terms of the treatment (a single-oral 400 mg dose of ALB originating from the same batch), the follow up (between 14 and 30 days after) and the detection technique (the McMaster counting technique). Moreover, efficacy was evaluated by both the CR and the FECR, and compared statistically between the seven trials which took place in geographically disparate parts of the world.

Overall, this study supports previous reports that indicated that single dose ALB treatment is most effective for infection with *A. lumbricoides*, followed by hookworm, but is relatively ineffective for *T. trichiura*, confirming the efficacy studies reviewed by Bennet and Guyatt [3], and by Keiser and Utzinger [8]. The low efficacy observed for *T. trichiura* compared to the two other STH, is in keeping with previous studies, where a 3-day dose schedule of ALB has been shown to be necessary to achieve acceptable therapeutic efficacy [3].

At present, the most commonly reported indicator of drug efficacy in this field is the CR [3]. Our results support the view that the CR should not be the recommended parameter, as it is sensitive to variation in the intensity of infection before treatment. The CRs declined in all three STH with increasing intensity of infection (FEC) at the pre-intervention survey. Hence, comparison between populations (countries, villages, schools, etc.) differing in pre-intervention FEC are guaranteed to arrive at different conclusions about drug efficacy. Differences in the outputs of calculations based on processing quantitative data in different ways also showed variation that requires careful review if standard operating procedures for data processing are to be adopted. The observation that therapeutic efficacies based on arithmetic means were mostly lower than those based on geometric means is in agreement with other studies [15], and arises because the arithmetic means captures the variation more effectively, while the geometric means compress the data such that efficacies are highly overestimated. Our exploratory analysis of different statistical approaches for analyzing data also indicates that FECR based on individuals was highly affected by excluding subjects with pre-intervention FEC below 150 EPG. Therefore, we conclude that the group based formula using an arithmetic mean is the best summary statistic to employ in analysis of therapeutic efficacy in future large scale drug administration trials, since it represents a robust indicator that is sensitive to changes in drug efficacy.

The efficacy (CR and FEC reduction rate) varied widely across the trials, except for *A. lumbricoides*. Possible explanations for the observed differences include (1) treatment history, (2) geographic differences within STH species, (3) fecal consistency and (4) diet. It is therefore pertinent to comment on each. Although the lowest efficacies for *T. trichiura* (Cameroon and Tanzania) and hookworms (India) were obtained in countries with a treatment history, the observed low efficacies are not likely to be attributable to large scale anthelmintic treatment in Cameroon and India. In these countries, a comparison between different study sites with a history of large scale anthelmintic treatment (Cameroon: Loum; India: Vellore) and without such a history (Cameroon: Yoyo; India: Thiruvanamalai) indicated that these large scale programs did not result in a reduced efficacy compared to sites were they were absent (data not shown and to be published separately). For Tanzania, the impact of large scale anthelmintic treatment programs could be ruled out, as studies before and during these interventions have shown similar drug efficacy figures for *T. trichuria* [16], [17].

Current molecular studies indicate that geographical differences exist within STH species [18], [19]. For *T. trichiura* varying anthelmintic efficacy has been suggested to be attributable to the presence/absence of the β-tubulin codon 200 polymorphism that has been linked to BZ resistance [20]. Strain differences have been demonstrated in some species with different drug tolerance as assessed both by efficacy and molecular studies [20], [21]. Nevertheless, the exact impact of genetic differences within the 3 STH in this study on the efficacy of specific anthelmintics remains speculative. Of note, even at a higher taxonomic level, information on the relative therapeutic efficacy of a single dose ALB on *N. americanus* and *A. duodenale* is scarce, this despite the distinct and well known biological differences between these hookworms [22]-[24]. FEC was calculated in the current study without compensation for fecal consistency. It is well recognized that well-formed stools can concentrate helminth eggs, compared to looser or diarrheic feces where they are diluted [25], thus confounding assessment of drug efficacy. Finally, the diet of subjects varied considerably across the seven participating countries. Differences in the quality of food consumed would have created differences in fat and roughage content and/or increased the rate of passage of substances through the gastrointestinal tract. This may have reduced the period over which ALB could have acted on the parasites, thereby reducing efficacy [26]-[28].

Kopp et al. [29] demonstrated that a reduction in adult canine hookworm (*A. caninum*) counts following chemotherapy did not always yield a reduction in FEC, due to an increase in fecundity among the small residual worm population that survived the anthelmintic treatment (i/e., density dependent fecundity), consequently confounding the FECR. As described by Kotze and Kopp [30], density dependent effects could be manifested in a FECR as a reduced drug efficacy for subjects with higher pre-intervention FEC. However, this did not occur in the present study for *A. lumbricoides* and hookworm. For *T. trichiura*, the efficacy did decrease with increasing pre-intervention FEC, but this should be interpreted with some caution. This effect was not consistent across the different trials (e.g., no correlation in Vietnam but a positive correlation in Tanzania), suggesting that other factors as discussed above may have confounded this result. It is also possible that increases in FEC may have arisen because of the inability of ALB to cure infections during the pre-patent period (with an onset of patency after the pre-intervention egg count time point). This is a complication that cannot be avoided in studies taking place in endemic areas where transmission occurs daily because of soil and food contaminated with infective stages of the parasites, and is not interrupted in the population during the period of study. Finally, a negative correlation between the FEC and efficacy is expected, as the probability of having a FEC of zero after treatment in the follow-up survey, consequently a FECR of 100%, will be higher for low FEC than for high FEC before the administration of the drug.

Our findings emphasize a need to adhere to strict standard operating procedures and methodologies, and to change the WHO recommended threshold levels for the efficacy of ALB [31], where a FEC reduction rate below 70% in the case of *A. lumbricoides* or below 50% for the hookworms are the currently accepted thresholds. We recommend that in future monitoring of single-dose ALB-dependent control programs a minimum FEC reduction rate (based on arithmetic means) of >95% for *A. lumbricoides* and >90% for hookworms are appropriate thresholds, and that efficacy levels below this should raise concern. The great variability of the FECR for *T. trichiura* and the relatively low efficacy of ALB, confirmed in this present study, indicate that it is not possible to propose an efficacy threshold for this parasite based on our data.

In conclusion, the present study is the first to evaluate drug efficacy of a single-oral dose of ALB on such a scale and across three continents. The results confirm the therapeutic efficacy of this treatment against *A. lumbricoides* and hookworms, and the low efficacy against *T. trichiura*. Efficacy varied widely across the seven different trials, particularly in the case of *T. trichiura* and it remains unclear which factors were principally responsible for this variation, although pre-intervention FEC and age played clear roles in this respect. The FEC reduction rate based on arithmetic means is the best available indicator of drug efficacy, and should be adopted in future monitoring and evaluation studies of large scale anthelmintic treatment programs. Finally, our findings emphasize the need to revise the WHO recommended efficacy threshold for single dose ALB treatments.

## Supporting Information

Checklist S1 CONSORT Checklist
(0.22 MB DOC)

Protocol S1 Trial Protocol
(1.17 MB PDF)

## References

[1] World Health Organization (2005). Deworming for health and development..

[2] Albonico M, Allen H, Chitsulo L, Engels D, Gabrielli AF (2008). Controlling soil-transmitted helminthiasis in pre-school-age
children through preventive chemotherapy.. PLoS Negl Trop Dis.

[3] Bennett A, Guyatt H (2000). Reducing intestinal nematode infection: efficacy of albendazole
and mebendazole.. Parasitol Today.

[4] Wolstenholme AJ, Fairweather I, Prichard R, von Samson-Himmelstjerna G, Sangster NC (2004). Drug resistance in veterinary helminths.. Trends Parasitol.

[5] Albonico M, Engels D, Savioli L (2004). Monitoring drug efficacy and early detection of drug resistance
in human soil-transmitted nematodes: a pressing public health agenda for
helminth control.. Int J Parasitol.

[6] Geerts S, Gryseels B (2001). Anthelmintic resistance in human helminths: a
review.. Trop Med Int Health.

[7] De Clercq D, Sacko M, Behnke JM, Gilbert F, Dorny P (1997). Failure of mebendazole in treatment of human hookworm infections
in the Southern Region of Mali.. Am J Trop Med Hyg.

[8] Keiser J, Utzinger J (2008). Efficacy of current drugs against soil-transmitted helminth
infections - systematic review and meta-analysis.. JAMA.

[9] Levecke B, De Wilde N, Vandenhoute E, Vercruysse J (2009). Field validity and feasibility of four techniques for the
detection of *Trichuris* in simians: a model for monitoring
drug efficacy in public health?. PLoS Negl Trop Dis.

[10] Coles GC, Bauer C, Borgsteede FH, Geerts S, Klei TR (1992). World Association for the Advancement of Veterinary Parasitology
(W.A.A.V.P.) methods for the detection of anthelmintic resistance in
nematodes of veterinary importance.. Vet Parasitol.

[11] Coles GC, Jackson F, Pomroy WE, Samson-Himmelstjerna G, Silvestre A (2006). The detection of anthelmintic resistance in nematodes of
veterinary importance.. Vet Parasitol.

[12] Ministry of Agriculture, Fisheries and Food (1986). Manual of veterinary parasitological laboratory techniques
(Reference Book; 418), 3rd ed..

[13] Montresor A, Crompton DWT, Hall H, Bundy DAP, Savioli L (1998). Guidelines for the evaluation of soil-transmitted helminthiasis
and schistosomiasis at community level..

[14] Crawley MT (1993). GLIM for Ecologists..

[15] Dobson RJ, Sangster NC, Besier RB, Woodgate RG (2009). Geometric means provide a biased efficacy result when conducting
a fecal egg count reduction test (FECRT).. Vet Parasitol.

[16] Albonico M, Smith PG, Hall A, Chwaya HM, Alawi KS (1994). A randomised controlled trial comparing mebendazole 500 mg and
albendazole 400 mg against *Ascaris, Trichuris* and the
hookworms.. Trans R Soc Trop Med Hyg.

[17] Albonico M, Bickle Q, Ramsan M, Montresor A, Savioli L (2003). Efficacy of mebendazole and levamisole alone or in combination
against intestinal nematode infections after repeated targeted mebendazole
treatment in Zanzibar.. Bull WHO.

[18] Hu M, Chilton NB, El-Osta YGA, Gasser RB (2003). Comparative analysis of mitochondrial genome data for
*Necator americanus* from two endemic regions reveals
substantial genetic variation.. Int J Parasitol.

[19] Leles D, Araújo A, Vicente AC, Iñiguez AM (2009). Molecular diagnosis of ascariasis from human feces and
description of a new *Ascaris* sp. genotype in
Brazil.. Vet Parasitol.

[20] Diawara A, Drake LS, Suswillo RR, Kihara J, Bundy DA (2009). Assays to detect B tubulin Codon 200 polymorphism in
*Trichuris trichiura* and *Ascaris
lumbricoides*.. PLoS Negl Trop Dis.

[21] Marti H, Haji HJ, Savioli L, Chwaya HM, Mgeni AF (1996). A comparative trial of a single-dose ivermectin versus three days
of albendazole for treatment of *Strongyloides stercoralis*
and other soil-transmitted helminth infections in children.. Am J Trop Med Hyg.

[22] Hoagland KE, Schad GA (1978). *Necator americanus* and *Ancylostoma
duodenale*: life history parameters and epidemiological
implications of two sympatric hookworms of human.. Exp Parasitol.

[23] El-Masry NA, Trabolsi B, Bassily S, Farid Z (1983). Albendazole in the treatment of *Ancylostoma
duodenale* and *Ascaris lumbricoides*
infections.. Trans R Soc Trop Med Hyg.

[24] Sacko M, De Clercq D, Behnke JM, Gilbert FS, Dorny P (1999). Comparison of the efficacy of mebendazole, albendazole and
pyrantel in treatment of human hookworm infections in the southern region of
Mali, West Africa.. Trans R Soc Trop Med Hyg.

[25] World Health Organization (1961). CCTA/WHO African conference on ancylostomiasis, Brazzaville
22–29 August 1961.. Technical Report Series No. 225.

[26] McKellar QA, Scott EW (1990). The benzimidazole anthelmintic agents. A review.. J Vet Pharmacol Ther.

[27] Dayan AD (2003). Albendazole, mebendazole and praziquantel. Review of non-clinical
toxicity and pharmacokinetics.. Acta Trop.

[28] Sanchez SF, Alvarez LI, Lanusse CE (1996). Nutritional condition affects the disposition kinetics of
albendazole in cattle.. Xenobiotica.

[29] Kopp SR, Coleman GT, McCarthy JS, Kotze AC (2008). Application of in vitro anthelmintic sensitivity assays to canine
parasitology: detecting resistance to pyrantel in *Ancylostoma
caninum*.. Vet Parasitol.

[30] Kotze AC, Kopp SR (2009). The potential impact of density dependent fecundity on the use of
the fecal egg count reduction test for detecting drug resistance in human
hookworms.. PLoS Negl Trop Dis.

[31] World Health Organization (1999). Report of the WHO informal consultation on monitoring of drug
efficacy in the control of schistosomiasis and intestinal
nematodes..

