# Supplementary material for: Assessment of the Anthelmintic Efficacy of Albendazole in School Children in Seven Countries Where Soil-Transmitted Helminths Are Endemic
Source: PLoS Negl Trop Dis. 2011 Mar 29;5(3):e948. doi: 10.1371/journal.pntd.0000948 (PMC3066140; doi:10.1371/journal.pntd.0000948)
Supplement: Protocol S1 — Trial Protocol (1.17 MB PDF) [file pntd.0000948.s002.pdf]

Protocol - Monitoring efficacy of anthelmintics for the treatment of Soil Transmitted Helminths (STH)

Prepared by

Jozef Vercruysse (coordinator), Antonio Montresor (WHO representative), Marco Albonico, Andrew Kotze, James McCarthy and Jerzy Behnke

Background & justification

The three major Soil-Transmitted Helminths of humans (STH's), *Ascaris lumbricoides* (roundworm), *Necator americanus/Ancylostoma duodenale* (the hookworms) *and Trichuris trichiura* (whipworm) are amongst the most prevalent parasites worldwide. The major means of controlling STH infections is by the periodic administration of one of the four anthelmintics recommended by the WHO: mebendazole (MBD), albendazole (ALB, the most common used anthelmintic to treat STH), levamisole & pyrantel. Currently, for the human STH, large scale mass chemotherapy campaigns are taking place in various parts of Africa, Asia and South America, particularly targeting school children. Although WHO recommends a parasitological check every few years in large scale treatment programmes, these are being conducted infrequently without a coherent long-term strategy and without a standardized monitoring protocol.

During a WHO-World Bank meeting (Washington, Nov 2007) it was decided that monitoring the efficacy of anthelmintics should be a key-issue and a Working Group was established to make recommendations for "Monitoring of drug efficacy in large scale treatment programmes for human STH".

Objectives

Overall Objective

The overall objective is to develop and validate a standard protocol to monitor efficacy of anthelmintics of Soil Transmitted Helminths (STH) in populations with different exposure to anthelmintics.

Primary objectives

1. To assess the change in Faecal Egg Counts (FEC) in school age children 14 days following the treatment with a single dose 400 mg of albendazole
2. To monitor efficacy by determining the Cure Rate (CR) and Egg Reduction Rate (ERR)

Secondary objectives

1. To evaluate the suitability of the Faecal Egg Count Reduction Test (FECRT) as a standard tool to monitor efficacy i.e. to develop a robust analytical approach that accounts for possible confounding factors
2. To compare the relative performance of the Kato Katz or other qualitative coprological techniques and the McMaster egg counting technique.

Study sites and population

Study sites

A total of 7 countries were selected by the working group: Brazil (Minas Gerais State), Cambodia (Kratie), Cameroon (Loum and Yoyo), Ethiopia (Jimma), India (Vellore, Thiruvanamalai), Tanzania (Zanzibar, Pemba Island), Vietnam (Thái Nguyên, Tuyên Quang). Selection criteria considered by the group in choosing the study sites were:

- The ability of the sites to perform the studies
  e.g. presence of equipped diagnostic facilities, skilled personnel
- A documented history of presence of STH
- A known history of drug selection pressure: high, low or none.

Study Population

Schoolchildren between 6 and 18 years old are the focus of this study because of two main reasons:

1. <u>Schoolchildren are the most accessible group</u>. In countries where school enrolment rates are good the group can be easily reach through the school system (Partnership For Child Development, 1999), in area where enrollment is not high non-enrolled siblings could be effectively outreached by promoting advocacy through the schools (Montresor *et al.*, 2001). Therefore it is considered that in each country where

resistance to benzimidazole is suspected, this group will be the easier one to be investigated.

2. Schoolchildren are the one that more probably could harbor resistant species of STH.
   Children of primary school age (6 - 18 years) are normally a major target for regular treatment with anthelminthic, because they are the group that usually has the heaviest worm burdens for *A. lumbricoides* and *T. trichiura*, and are steadily acquiring hookworm infections. In addition, they are in a period of intense physical and intellectual growth (Bundy *et al.* 1992; Crompton and Nesheim, 2002). Deworming school-age children has a considerable benefit on their nutritional status (Stoltzfus *et al.*, 1996, Curtale *et al.*, 1995), physical fitness, appetite, growth (Stephenson *et al.*, 1993) and intellectual development (Nokes et al 1994).

Ethical issues

The overall protocol of the study has been reviewed by the Ethics committee of the Faculty of Medicine, Ghent University, Belgium on 15/07/2008. A positive advice was given on 30/06/2008 (Nr B67020084254) (Annex 1). A sample of consent form and the accepted protocol is also attached.

For each proposed study a separate ethical clearance will be obtained.

Parasitological technique to determine FEC

The standard coprological technique to be used will be the McMaster method, a user-friendly method widely practiced in Veterinary Medicine. The McMaster method is a quantitative method for analysis of FEC. For each sample one chamber (two compartiments) will be counted (sensitivity of 50 eggs/gram) (Annex 2.1). A video showing the McMaster technique will be made and provided to all participating laboratories.

McMaster slides will be provided free by the coordinating group

Preliminary Comparative study of the coprological techniques

As there has been relatively relative little use of the McMaster method for human coprologic examination, a pilot study comparing the Kato Katz (Annex 2.2) and McMaster method (Annex 2.1) should be undertaken on 200 samples at each study site. From the 200 samples at least 100 samples should be positive for STH in either technique at each study site.

As the Kato Katz method has an estimated sensitivity threshold of 24 eggs/gram of faeces, two McMaster chambers (4 compartiments, sensitivity of 25) will be counted for the comparative study.
In the case that the local routine coprological technique is not the Kato Katz , the McMaster method should be compared with the routine technique in use.

Efficacy study

<u>Experimental design</u>

Following obtaining informed consent, schoolchildren in the target age range group will be recruited and asked to provide a recent stool sample (an interval of less that 4 hours) that will be processed to determine the FEC for each STH present. A small subsample of faecal samples will be also collected for coproculture to identify the hookworms species present and preserve parasite material for subsequent analysis (Annex 2.3).

All children providing stool samples will be treated with Albendazole (GSK®) single table of 400mg under supervision (chewing + water). The Albendazole will be provided (free) by the coordinating group.

14 to 30 days after treatment a second faecal sample will be collected from the children to determine again FEC. Only children infected with a minimum of 150 eggs/gram of any species of STH (i.e. *Ascaris* or hookworms or *Trichuris*) at the first sampling should be included in the second sampling. However, for the other STH present FEC should also be determined at the second sampling.

Sample size

The initial number of schoolchildren to sample will depend on the prevalence of the STH in the study region as a minimum of 250 infected children[1] with a minimum of 150 eggs/gram of any species of STH (i.e. *Ascaris* or hookworms or *Trichuris*) should be recruited at each study site e.g. with an expected prevalence of 50% for any specific STH 500 schoolchildren should be enrolled in the first sampling.

If the epidemiological scenario allows, individual study sites may include two groups of children (each with 250 infected children with a minimum of 150 eggs/gram of any species of STH): (1) a group of children resident in areas where the government had, for ≥ than 3 years, a regular (one or two treatments/year) mass drug administration program with a benzimidazole anthelmintic (albendazole or mebendazole) and (2) a group of children resident in areas where there is no mass drug administration program.

Exclusion Criteria:

- Not willing to participate
- Unable to give samples for follow up
- Severe intercurrent medical condition
- Diarrhoea at first sampling

Data analysis

Both CR and ERR will be considered to monitor to efficacy.

To avoid distortion due to the density dependant effect, the CR and ERR will be also calculated separately in the different classes of intensity at baseline.

[1] Statistical power analyses, based on random simulations of correlated overdispersed FEC data reflecting the variance-covariance structure in a selection of real FEC data sets, suggest that a sample size of up to 200 individuals ($\alpha = 0.05$, power = 80%) is required to detect a 10 percentage point drop from a null efficacy of ~ 80% (mean percentage FEC Δ per individual) over a wide range of infection scenarios. Standard power analyses for proportions also indicate that the detection of a ~10 percentage point drop from a null cure rate requires sample sizes ranging up to 200 (the largest samples being required to detect departures from null efficacies around 50%). Given an anticipated non-compliance rate of 25%, a sample of 250 individuals with >250 egg/g pre-treatment should therefore be followed up for post-treatment FEC data at each study locality.

The following indexes should be used to monitor drug efficacy:

Cure rate

[N egg positive post-treatment / N egg positive pre-treatment]*100

Mean FEC Δ per individual

$$\left(\sum_{i=1}^{i=N} [T1_i - T2_i] \right) / N$$

Where T1 = pre-treatment FEC, T2 = post-treatment FEC, and *i* = the *i*th subject.

Mean percentage FEC Δ per individual

$$\left(\sum_{i=1}^{i=N} \{[T1_i - T2_i] / T1_i \}*100 \right) / N$$

Percentage Δ in the sample mean FEC

{[sample mean T1- sample mean T2] / sample mean T1 }*100

Confidence intervals for these indexes can be generated by a standard method for proportions in the case of cure rate (the exact binomial method) and by bootstrap resampling methodologies for the FEC Δ indexes.

References

Bundy, D.A., Hall, A., Medley, G.F. and Savioli, L. (1992). Evaluating measures to control intestinal parasitic infections. *World Health Statistics Quarterly* **45**, 168-179.

Crompton, D.W.T. and Nesheim, M.C. (2002) Nutritional impact of intestinal helminthasis during the human life cycle. *Annual Review of Nutrition* **22,** 35-59.

Curtale, F., Pokhrel, R.P., Tilden, R.L. and Higashi, G. (1995) Intestinal helminths and xerophthalmia in Nepal. A case-control study. *Journal of Tropical Pediatrics* **41**, 334-337.

Montresor, A., Ramsan, M, Chwaya, H.M., Ameir, H., Foum, A., Albonico, M., Gyorkos, T.W. and Savioli, L. (2001). Extending anthelminthic coverage to non-enrolled school-age

children using a simple and low-cost method. *Tropical Medicine and International Health* **6**, 535-537.

Nokes, C. and Bundy, D.A. (1994). Does helminth infection affect mental processing and educational achievement? *Parasitology Today* **10**, 14-18.

Partnership for Child Development (1999). The cost of large-scale school health programmes which deliver anthelminthics to children in Ghana and Tanzania. *Acta Tropica* **73,** 183 - 204.

Stephenson, L.S., Latham, M.C., Adams, E.J., Kinoti, S.N. and Pertet, A. (1993). Physical fitness, growth and appetite of Kenyan school boys with hookworm, *Trichuris trichiura* and *Ascaris lumbricoides* infections are improved four months after a single dose of albendazole. *Journal of Nutrition* **123**, 1036-1046.

Stoltzfus, R.J., Albonico, M., Chwaya, H.M., Savioli, L., Tielsch, J., Schulze, K. and Yip, R. (1996). Hemoquant determination of hookworm-related blood loss and its role in iron deficiency in African children. *American Journal of Tropical Medicine and Hygiene* **55**, 399-404.

Annex 1
Consent Form – Ethical issues

The advice of the Ethics committe (B67020084254), Faculty of Medicine, Ghent University (Belgium) and the corresponding protocol are attached

Consent Form

Albendazole against worm infections in …….children

We are conducting a study to find the best way of treating children in ……. for intestinal worms. Intestinal worms are very common here and make children anaemic and reduce their growth. You may know there is a National Control Programme in …… that distributes deworming tablets and to all children in schools every six months.

Doctors from the Ministry of Health, and from The World Health Organization (Geneva), need to carry out a study to see how well the deworming drugs are working to cure the worms infections in ……. children. The drug is called Albendazole and it is given in the form of a tablet that children chew with a drink of water. We are asking children aged between 5-14 years from some schools to participate. The school your child is going to has been one of the schools chosen for this study.

After agreeing that your child can take part, one of the research staff will visit the school on a certain day and give your child a small plastic container and ask him/her to produce a sample of his/her faeces. The plastic container with the stool will be collected the next morning in the school.

We will check the stool to see if your child has worms and we will provide him/her treatment. This will make your child healthier.
Sometimes a child may experience a minor side effect after taking a tablet of albendazole. These effects, if they occur, include headache, feeling dizzy, itching and nausea. They last for a short time and disappear by themselves. In case of any persistent problem you are advised to refer your child to the nearest Health Centre where staff has been trained and medication are available for this purpose.

After 14 days, we will ask your child to give another faeces sample to see how well the drug has worked against the worms. The faecal samples positives with worms will be stored in the laboratory in case other tests need to be carried out. At the end of the study they will be disposed of by a safe procedure.

Some information about your child will be recorded on anonymous forms that will be kept in a locked room at the office in ……….. Only the researchers doing the study will use these forms.

If you have any questions about the study at any time, you can call the Project Manager
Dr ………………… at……………..., (Phone ………………..).

After the study, all the results of the deworming study will be presented in a public meeting in your child's school by Ministry of Health staff.

We assure you that our best care will be taken of your child if you agree to let him/her take part in the study. You should also know that you are free to withdraw your child from the study at any time and that he/she will not be discriminated in any form for education or health services.

I have read the information above, or it has been read to me. I have been given the opportunity to ask questions and my questions have been answered to my satisfaction. I voluntarily consent that my child participates in this study.

I agree to enroll my child in this study:

Date…………… Signature………………………………………

Ethical considerations

Ethical Committee Review

An overall Ethical clearance for the study was obtained from the Human Research Ethics Committee of the University of Ghent, is submitted at the Queensland Institute for Medical Research, and from the respective research ethics committees of the 6 Countries (Brazil, Cameroun, India, Vietnam, Zanzibar & Ethiopia) involved in this multicentric study.

Informed consent and enrolment

The Principal Investigators will prepare an Informed Consent Document and submit it to National research ethics committees of the respective countries, and to the Human Research Ethics Committee of the University of Ghent for approval. The consent form will be written in English and translated into each of the local language (sample attached in the Annex). Prior to the trial in the selected study sites, public meetings will be held with the parents/guardians, community leaders and teachers, where the purpose of the trial, safety precautions and benefits for the study population will be explained by qualified study staff. It will be emphasized that participation in the trial is entirely voluntary; neither the parents/guardians nor their children will be discriminated against in any way if they choose not to enter their children into the trial. Prior to agreement to enrol their children in the trial, parents/guardians will be given every opportunity to have their questions answered. Then the parents/guardians will be asked to sign the informed consent document. After signing, one copy of the document will be given to the parent/guardian, while the other will be brought to the study site on the day of enrolment to verify that consent has been given.

Confidentiality

The information obtained during the conduct of this study will remain confidential. Disclosure of any of the data to third parties other than those allowed in the Informed Consent form will not be permitted. The results of the research study may be published, but subjects' names or identities will not be revealed. Records will remain confidential. To maintain confidentiality, the Senior Scientist will keep records in locked cabinets and the results of the tests will be coded to prevent identification of the volunteers. Access to data entered into computerized files will permitted only for authorized personnel directly involved with the study and will be password protected. Subject-specific information may be provided to responsible local medical personnel only with the subject's permission. Stool samples collected will not be used for research purposes other than those outlined in the protocol, and will be safely disposed of after the completion of the study.

Feed-back after the trial has been completed

At the end of the trial, the results of the study will be communicated to the Ministry of Health (MoH) of each study site. In addition, the MoH and Ministry of Education (MoE) officers in charge of the districts where the study had been performed will inform the community of the results through the most appropriate channel, for example, a newsletter or a public meeting. The Department of Control of Neglected Tropical Disease at WHO, Geneva, will coordinate the international dissemination of the study results, including publication.

Safety

This trial will only use original albendazole product that has already been approved by the National Regulatory authority of each country in which the trial will be carried out. It is recognized that all study sites have been regularly using albendazole or mebendazole in National mass deworming campaigns. The drugs will only be administered by qualified health professionals as outlined in the study protocol. Although treatment with albendazole

has been ongoing as a Public Health intervention, and the safety of the drug in the study group has been well demonstrated, referral to appropriate medical professionals will be made available for any rare but possible severe adverse event occurring during the study will be provided by the study team.

Benefits

Children participating in the trial will directly benefit by being investigated for STH infections and receiving appropriate treatment. Children still infected at the end of the observation period will immediately be offered additional treatment according of their specific clinical situation. The study results will help to guide WHO and the international scientific community in choosing the most effective protocol to monitor anthleminthic efficacy, and will also guide global recommendations for STH endemic countries.

UZ Universitair Ziekenhuis Gent
Afz: Commissie voor Medische Ethiek

UNIVERSITEIT GENT
COMMISSIE VOOR MEDISCHE ETHIEK

Voorzitter:
Prof. Dr. R. Rubens
Secretaris:
Prof. Dr. D. Matthys

Klinische Biologie, Microbiologie en Immunologie
Behandelingsblok A - 1ste Verdieping
Prof. dr. Jozef VERCRUYSSE
ALHIER

CONTACT	TELEFOON	FAX	E-MAIL
Secretariaat	+32 (0)9 332 56 13<br>+32 (0)9 332 59 25	+32 (0)9 332 49 62	ethisch.comite@ugent.be
**UW KENMERK**	**ONS KENMERK**	**DATUM**	**KOPIE**
	2008/322	30-jun-08	Zie "CC"

BETREFT
Advies voor monocentrische studie met als titel:
Monitoring the efficacy of anthelmintics for the treatment of soil transmitted helminths.

Belgisch Registratienummer: B67020084254

* Antwoord onderzoeker ontv. 27/06/2008 op opmerkingen EC dd. 25/06/2008
* Adviesaanvraagformulier dd. 23/06/2008
* (Patiënten)informatie- en toestemmingsformulier (aangepaste versie ontv. 26/06/2008)

Advies werd gevraagd door:
Prof. dr. J. VERCRUYSSE ; Hoofdonderzoeker

BOVENVERMELDE DOCUMENTEN WERDEN DOOR HET ETHISCH COMITÉ BEOORDEELD.
ER WERD EEN POSITIEF ADVIES GEGEVEN OVER DIT PROTOCOL OP 30/06/2008

THE ABOVE MENTIONED DOCUMENTS HAVE BEEN REVIEWED BY THE ETHICS COMMITTEE.
A POSITIVE ADVICE WAS GIVEN FOR THIS PROTOCOL ON 30/06/2008

DIT ADVIES WORDT OPGENOMEN IN HET VERSLAG VAN DE VERGADERING VAN HET ETHISCH COMITE VAN 15/07/2008

THIS ADVICE WILL APPEAR IN THE PROCEEDINGS OF THE MEETING OF THE ETHICS COMMITTEE OF 15/07/2008

- *Het Ethisch Comité werkt volgens 'ICH Good Clinical Practice' - regels*
- *Het Ethisch Comité beklemtoont dat een gunstig advies niet betekent dat het Comité de verantwoordelijkheid voor het onderzoek op zich neemt.*
- *In het kader van 'Good Clinical Practice' moet de mogelijkheid bestaan dat het farmaceutisch bedrijf en de autoriteiten inzage krijgen van de originele data. In dit verband dienen de onderzoekers erover te waken dat dit gebeurt zonder schending van de privacy van de proefpersonen.*
- *Het Ethisch Comité benadrukt dat het de promotor is die garant dient te staan voor de conformiteit van de anderstalige informatie- en toestemmingsformulieren met de nederlandstalige documenten.*
- *Geen enkele onderzoeker betrokken bij deze studie is lid van het Ethisch Comité.*
- *Alle leden van het Ethisch Comité hebben dit project beoordeeld. (De ledenlijst is bijgevoegd)*

- *The Ethics Committee is organized and operates according to the 'ICH Good Clinical Practice' rules.*
- *The Ethics Committee stresses that approval of a study does not mean that the Committee accepts responsibility for it.*
- *In the framework of 'Good Clinical Practice', the pharmaceutical company and the authorities have the right to inspect the original data. The investigators have to assure that the privacy of the subjects is respected.*
- *The Ethics Committee stresses that it is the responsibility of the promotor to guarantee the conformity of the non-dutch informed consent forms with the dutch documents.*
- *None of the investigators involved in this study is a member of the Ethics Committee.*
- *All members of the Ethics Committee have reviewed this project. (The list of the members is enclosed)*

Namens het Ethisch Comité / On behalf of the Ethics Committee

Prof. dr. R. RUBENS
Voorzitter / Chairman

CC: UZ Gent - Beheer en algemene directie
FAGG - Research & Development; Victor Hortaplein 40, postbus 40 1060 Brussel

Annex 2

Parasitological techniques

Annex 2.1 - McMaster technique

The McMaster technique uses a counting chamber which enables a known volume of faecal suspension (2 x 0.15 ml) to be examined microscopically. Thus, if a known weight of faeces and a known volume of flotation fluid are used to prepare the suspension, then the number of eggs per gram of faeces (FEC) can be calculated.
The quantities are chosen so that the faecal egg-count can be easily derived by multiplying the number of eggs under the marked areas by a simple conversion factor.
The McMaster chamber has two compartments, each with a grid etched onto the upper surface. When filled with a suspension of faeces in flotation fluid, much of the debris will sink while eggs float to the surface, where they can easily be seen and those under the grid counted.

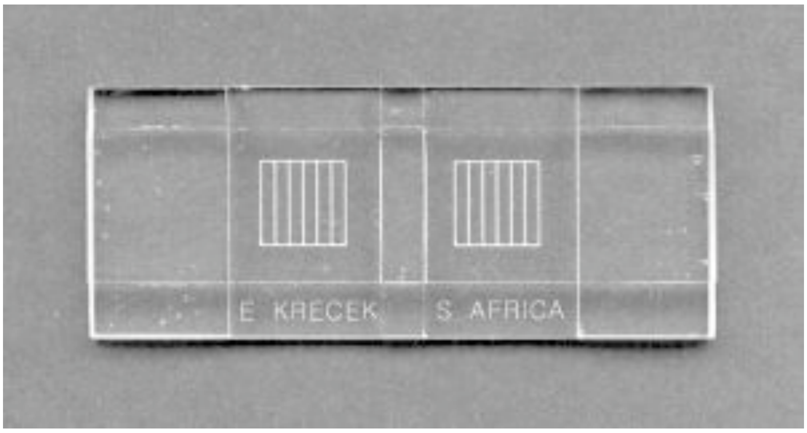

Equipment List

* Two beakers or plastic containers
* Balance
* Tea strainer
* Measuring cylinder
* Stirring device (fork, spatula, tongue depressor)
* Pasteur pipettes and rubber teats
* Flotation fluid (density of 1.22): to be prepared at least one day before use and kept at temperatures > 20°C. The flotation fluid is a saturated salt solution, with a minimum of 333 g NaCl in 1L of aq dest; the presence of a salt sedimention confirms saturation
* McMaster counting chamber
* Compound microscope

Procedure

1. Weigh 2 grams of faeces in a plastic beaker (100 ml).
2. Add 30 ml of the flotation fluid saturated salt solution,
3. Homogenise and pour the faecal suspension 3 times through a tea strainer to withhold the large debris.
4. Homogenise the filtrate by pouring it at least 5 times from one beaker to another and fill up one side of a regular McMaster counting chamber by using a pasteur pipette. Repeat for the other side.
5. Allow the counting chamber to stand for 2 minutes, place under a light microscope and examine using a 100x magnification. All the eggs under the two separate grids are counted (this represents a volume of 2x 0.15 ml)
6. The number of eggs per gram of faeces is obtained by multiplying the total number of eggs under the two grids by 50. The sensitivity of the test is 50.

Annex 2.2 - Kato-Katz technique-cellophane faecal thick smear

Materials and reagents

1. Applicator sticks, wooden.
2. Screen, stainless steel, nylon or plastic 60-105 mesh.
3. Template, stainless steel, plastic, or cardboard . Templates of different sizes have been produced in different countries. A hole of 9 mm on a 1 mm thick template will deliver 50 mg of faeces; a hole of 6 mm on a 1.5 mm thick template, 41.7 mg; and a hole of 6.5 mm on a 0.5 mm thick template, 20 mg. The templates should be standardized in the country and the same size of templates should always be used to ensure repeatability and comparability of prevalence and intensity data.
4. Spatula, plastic.
5. Microscope slides (75 x 25 mm).
6. Hydrophilic cellophane, 40-50 mm thick, strips 25 x 30 or 25 x 35 mm in size.
7. Flat-bottom jar with lid.
8. Forceps
9. Toilet paper or absorbent tissue.
10. Newspaper.
11. Glycerol-malachite green or glycerol-methylene blue solution (1 ml of 3% aqueous malachite green or 3% methylene blue is added to 100 ml of glycerol and 100 ml of distilled water and mixed well). This solution is poured onto the cellophane strips in a jar and left for at least 24 h prior to use.

Procedure

1. Place a small mound of faecal material on newspaper or scrap paper and press the small screen on top so that some of the faeces are sieved through the screen and accumulate on top.
2. Scrape the flat-sided spatula across the upper surface of the screen to collect the sieved faeces.
3. Place template with hole on the centre of a microscope slide and add faeces from the spatula so that the hole is completely filled. Using the side of the spatula pass over the template to remove excess faeces from the edge of the hole (the spatula and screen may be discarded or, if carefully washed, may be reused).
4. Remove the template carefully so that the cylinder of faeces is left on the slide.
5. Cover the faecal material with the pre-soaked cellophane strip. The strip must be very wet if the faeces are dry and less so if the faeces are soft (if excess glycerol solution is present on upper surface of cellophane wipe with toilet paper). In dry climates excess glycerol will retard but not prevent drying.
6. Invert the microscope slide and firmly press the faecal sample against the hydrophilic cellophane strip on another microscope slide or on a smooth hard surface such as a piece of tile or a flat stone. The faecal material will be spread evenly between the microscope slide and the cellophane strip. It should be possible to read newspaper print through the smear after clarification.
7. Carefully remove slide by gently sliding it sideways to avoid separating the cellophane strip or lifting it off. Place the slide on the bench with the cellophane upwards. Water evaporates while glycerol clears the faeces.
8. For all except hookworm eggs, keep slide for one or more hours at ambient temperature to clear the faecal material prior to examination under the microscope. To speed up clearing and examination, the slide can be placed in a 40°C incubator or kept in direct sunlight for several minutes.

9. *Ascaris* and *Trichuris* eggs will remain visible and recognizable for many months in these preparations. Hookworm eggs clear rapidly and will no longer be visible after 30-60 minutes. Schistosome eggs may be recognizable for up to several months but it is preferable in a schistosomiasis endemic area to examine the slide preparations within 24 hours.
10. The smear should be examined in a systematic manner and the number of eggs of each species reported. Later multiply by the appropriate number to give the number of eggs per gram of faeces (by 20 if using a 50 mg template; by 50 for a 20 mg template; and by 24 for a 41.7 mg template). With high egg counts, to maintain a rigorous approach while reducing reading time, the Stoll quantitative dilution technique with 0.1 mol/litre NaOH may be recommended (see *Basic laboratory methods in medical parasitology,* WHO, 1991).

Annex 2.3 – Coproculture technique

To isolate infective stage hookworm larvae, feces are mixed with sufficient volume of water to enable homogenization by shaking in a sealed two liter container.
An equal volume of vermiculite is then mixed with the faecal suspension.
Further water is added until the mixture is uniformly moist. The mixture is incubated at 25-30°C for 5-7 days, being stirred daily.
[WEAR GLOVES FROM HERE ON] After five days, the mixture is spread onto large glass dishes such as Petri dishes, covered with a 5mm layer of washed, coarse river sand and overlaid with two layers of damp surgical gauze. The top layer of gauze (into which infective L3 migrate) is removed (and replaced) at 12 hour intervals, to be rinsed in distilled water. Larvae are retrieved from the suspension by gravitational sedimentation.

Optional: Viable larvae can be stored in BU buffer (50mM Na_2HPO_4, 22mM KH_2PO_4, 70mM NaCl) at 12°C for some time to preserve their infectivity (Hawdon 1991). To reduce bacterial and fungal contamination amphotericin B (Fungizone BristolMeyersSquib 0.25µg.ml µg/ml) and ceftriaxone (Rocephin Roche Y 20 µg/ml can be added at the start of the culture.
